# Enhancing Proton Therapy Efficacy Through Nanoparticle-Mediated Radiosensitization

**DOI:** 10.3390/cells13221841

**Published:** 2024-11-07

**Authors:** Jie Ma, Hao Shen, Zhaohong Mi

**Affiliations:** Key Laboratory of Nuclear Physics and Ion-Beam Application (MOE), Institute of Modern Physics, Fudan University, Shanghai 200433, China

**Keywords:** proton therapy, nanoparticle radiosensitization, RBE, cancer cells

## Abstract

Proton therapy, characterized by its unique Bragg peak, offers the potential to optimize the destruction of cancer cells while sparing healthy tissues, positioning it as one of the most advanced cancer treatment modalities currently available. However, in comparison to heavy ions, protons exhibit a relatively lower relative biological effectiveness (RBE), which limits the efficacy of proton therapy. The incorporation of nanoparticles for radiosensitization presents a novel approach to enhance the RBE of protons. This review provides a comprehensive discussion of the recent advancements in augmenting the biological effects of proton therapy through the use of nanoparticles. It examines the various types of nanoparticles that have been the focus of extensive research, elucidates their mechanisms of radiation sensitization, and evaluates the factors influencing the efficiency of this sensitization process. Furthermore, this review discusses the latest synergistic therapeutic strategies that integrate nanoparticle-mediated radiosensitization and outlines prospective directions for the future application of nanoparticles in conjunction with proton therapy.

## 1. Introduction

Radiotherapy plays a vital role in cancer treatment, benefiting over 50% of patients diagnosed with the disease [1]. Advanced techniques in radiotherapy, which employ X-rays or gamma rays, enable the precise targeting of tumors while delivering high doses of radiation directly to the affected area [2]. However, the collateral damage these rays cause to healthy tissues surrounding the tumor and along the radiation pathway is considerable, leading to side effects that can negatively impact the patient’s quality of life [3]. Unlike X-rays, which progressively lose energy as they traverse through matter upon entering the body, energetic protons or heavy ions predominantly deposit most of the energy at the end of their ranges, known as the Bragg peak [4]. The depth–dose distribution of protons is highly conformal, allowing for the maximum destruction of cancer cells while sparing healthy tissues, thereby achieving a superior therapeutic ratio compared to traditional photon therapy [5,6]. Nonetheless, the relative biological effectiveness (RBE) of protons, approximately 1.1, remains significantly lower than that of heavy ions such as carbon ions, which exhibit an RBE ranging from 2 to 5 [7,8]. This disparity underscores the necessity for further enhancements in the efficacy of proton therapy. A promising strategy involves the incorporation of nanoparticles as radiosensitizers to enhance the killing of cancer cells during proton therapy.

Nanoparticles, characterized by dimensions less than 100 nm [9], possess a high surface-to-volume ratio and exhibit effective penetration capabilities within deep tissue [10]. Moreover, owing to the enhanced permeability and retention (EPR) effect, these nanoparticles tend to accumulate in tumor tissues at elevated concentrations following either intravenous or direct tumor injection, thereby demonstrating superior tumor-targeting properties [11]. These attributes, coupled with favorable biocompatibility, represent the primary advantages of utilizing nanoparticles in radiation therapy. Numerous studies have demonstrated that nanoparticles can function as potent radiosensitizers, facilitating increased dose deposition in irradiated cancer cells during radiotherapy, which results in enhanced cancer cell lethality and improved therapeutic efficacy [12,13,14]. Among the various types of nanoparticles investigated [15,16], high-atomic-number (high-*Z*) nanoparticles have gained considerable attention due to their remarkably high interaction cross-sections with X-ray photons [17]. Examples of such high-*Z* nanoparticles include gold, platinum, gadolinium, and so on [18].

In the context of proton therapy, however, the Coulomb interaction between charged particles and nanoparticles exhibits limited dependence on the atomic number (*Z*), suggesting that nanoparticles are not theoretically expected to produce distinct radiosensitization effects [19,20]. Nevertheless, preliminary in vitro studies conducted by Liu et al. revealed that gold nanoparticles could enhance the biological effectiveness of proton therapy, as evidenced by a considerable reduction in the survival fraction of EMT-6 and CT26 cell lines [21]. Subsequently, a variety of simulations and in vivo and in vitro experiments have been carried out to investigate the radiosensitization effects of nanoparticles in proton therapy and to elucidate the underlying mechanisms [22,23]. This therapeutic approach shows potential for further increasing the RBE of proton therapy and improving the eradication of cancer cells.

This review aims to assess the current state of research regarding nanoparticles as radiosensitizers in proton therapy, summarizing the types of nanoparticles that exhibit superior radiosensitization effects. Furthermore, based on the existing experimental and simulation data, the mechanisms and influencing factors associated with nanoparticle radiosensitization are discussed, providing insights for future advancements in proton therapy techniques.

## 2. Nanoparticles Used for Proton Radiosensitization

**Figure 1:**
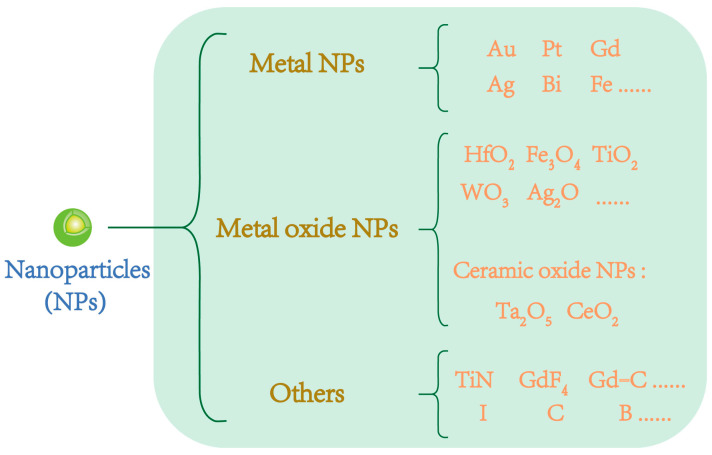
Classification of nanoparticles (NPs) with potential radiosensitization effects in proton therapy.

A range of nanoparticles have been examined for their potential radiosensitization effects in proton therapy, and the findings are summarized in Figure 1 [24]. Among these, the most extensively researched nanoparticles, which have shown distinct advantages in experimental or simulation studies, can be categorized into several groups: metal nanoparticles, metal oxide nanoparticles including several ceramic oxides, and other types of nanoparticles.

### 2.1. Metal Nanoparticles

Metal nanoparticles have a significant radiosensitization effect in radiotherapy with X-rays due to their high atomic number and adjustable surface chemical characteristics [25,26]. Several investigations have demonstrated that these nanoparticles can also enhance the physical dose deposition and thus amplify radiobiological effects in proton therapy [22]. Here, the metal nanoparticles currently under investigation as potential radiosensitizers for proton therapy are discussed.

#### 2.1.1. Gold Nanoparticles

Gold nanoparticles are widely recognized as one of the most promising metal nanoparticles in the field of radiation sensitization research despite their relatively high cost [27,28]. Their advantageous properties, including high electron density, ease of synthesis, variable sizes, customizable surface functionalization, low cytotoxicity, effective tumor targeting, and chemical stability, render them ideal candidates for use as radiosensitizers in proton therapy [29,30]. The preliminary in vitro experiments conducted by Liu et al. documented the sensitization effect of gold nanoparticles in proton therapy [21]. Following this, Lin et al. developed a Monte Carlo model to analyze the radiosensitization effects of gold nanoparticles under proton irradiation and predicted a significant nanoscale dose enhancement [31]. Polf et al. demonstrated that the internalization of gold nanoparticles by cells resulted in a 15–20% reduction in the survival of prostate cancer cells following proton irradiation in vitro [32]. Subsequently, Kim et al. conducted in vivo experiments using Balb-c mice injected with CT26 cancer cells to confirm the tumor growth inhibition effect enhanced by gold nanoparticles [33,34]. Their findings indicate that there was a remarkable reduction in tumor volume growth when gold nanoparticles were used in conjunction with proton irradiation compared to mice that only received proton treatment. Furthermore, a simulation study by Tran et al. illustrated that proton irradiation not only increases dose deposition around spherical gold nanoparticles but also significantly enhances the production of radiolysis due to the generation of secondary electrons, leading to an increase in cytotoxic radical species [35]. As a result, the radiosensitization effects of gold nanoparticles in proton therapy have garnered increasing interest, prompting numerous simulation and experimental studies aimed at further validating their sensitization effects and elucidating the underlying mechanisms [36,37,38,39,40,41,42].

In recent years, advancements in comprehensive physical dose models and biological models have been made to explore the radiosensitization effects of gold nanoparticles in proton therapy. Velten et al. employed the simulation tools TOPAS (version 3.8.p1 built against Geant4 10.07.p3) and Geant4-DNA to analyze the enhanced macro- and micro-dose depositions associated with gold nanoparticles, respectively, and developed a bio-model to assess the survival fraction of MDA-MB-231 breast cancer cells [43]. Their finding regarding the radiosensitization effects of gold nanoparticles was consistent with that of Lin et al., thereby reinforcing the evidence for increased dose deposition and biological damage attributed to gold nanoparticles in the context of proton therapy. Furthermore, Rajabpour et al. conducted an evaluation of the impact of various physical interaction models on the enhanced dose deposition and radiochemical yield of gold nanoparticles subjected to proton irradiation [44]. Their study demonstrated a similarity of within 15% between the Livermore and Penelope models available in Geant4, thereby establishing reliable frameworks for subsequent simulation studies investigating the mechanisms of gold nanoparticle-enhanced proton therapy. Additionally, Ahn compared the dose enhancement effects of gold nanoparticles when irradiated with protons, helium ions, and carbon ions [45]. The calculated dose enhancement ratio (DER) at a distance of 1 nm from the surface of the gold nanoparticles indicated that these nanoparticles could serve as effective radiosensitizers in ion-beam therapy, with protons exhibiting the most significant dose enhancement effect, followed by helium ions and carbon ions.

Recent investigations have demonstrated that gold nanoparticles can significantly enhance biological effects in both in vitro and in vivo settings. Cunningham et al. conducted in vitro studies on CHO-K1 cells, revealing the radiosensitization effect of gold nanoparticles when exposed to 200 MeV protons, which resulted in a statistically significant increase in cell mortality among those cells that had internalized the nanoparticles [46]. Zwiehoff et al. developed a fluorescence-based methodology to assess the generation of reactive oxygen species (ROS) induced by gold nanoparticles during proton irradiation [47]. Their findings indicate that the elevated ROS production associated with gold nanoparticles contributed to apoptosis and augmented indirect damage in proton therapy, thereby suggesting a substantial potential for enhancing the efficacy of this therapeutic approach. Furthermore, Johny et al. found that the combined effects of gold nanoparticles and proton therapy could arrest the G2 phase of the cell cycle in human medulloblastoma cells, leading to a significant impairment of the cells’ proliferation [48].

#### 2.1.2. Platinum Nanoparticles

Platinum-based materials, characterized by their high atomic number, are frequently employed as chemotherapeutic agents and potential diagnostic agents [49]. Notably, platinum nanomaterials have been shown to possess intrinsic anticancer properties, which can lead to increased DNA damage and enhanced cellular apoptosis [50]. These nanomaterials are capable of inducing immunogenic cell death, thereby activating the immune system to identify and target cancerous cells. In a study conducted in 2011, Porcel et al. explored the application of platinum nanoparticles as radiosensitizers in ion-beam therapy [51]. Their findings indicate that the energy deposition in proximity to the platinum nanoparticles was significantly enhanced due to an amplified electron cascade occurring within the nanoparticles during carbon ion irradiation, which, in turn, resulted in increased lethal damage to DNA. In further investigations, the DNA damage induced by platinum nanoparticles under different types of ionizing radiation was compared, revealing that these nanoparticles effectively amplify molecular damage when subjected to both photon and ion irradiation [52]. In a simulation study examining dose enhancement associated with different high-*Z* elements, Wälzlein et al. discovered that the local dose enhancement surrounding platinum nanoparticles under proton irradiation surpassed that of gold nanoparticles, highlighting platinum’s considerable potential to augment the radiobiological effects of proton therapy [53]. Additionally, Schlathölter et al. provided the first evidence of increased nanoscale damage induced by platinum nanoparticles under proton irradiation, utilizing DNA molecular probes to attribute this effect to the increased production of radical species [54].

Recently, platinum nanoparticles have demonstrated significant radiosensitization effects in radiotherapy. For instance, Batooei et al. evaluated the radiation dose effects and hydrolysis byproducts of commonly used high-*Z* nanoparticles under 6 MV X-ray irradiation utilizing the Genant4-DNA Monte Carlo simulation method. Their study further simulated the radiosensitization effects of platinum nanoparticles on gastric adenocarcinoma cells, thereby establishing that platinum nanoparticles can serve as potent radiosensitizers [55,56]. Motivated by the effective sensitization properties of platinum nanoparticles in X-ray radiotherapy, there has been growing interest in investigating their radiosensitization capabilities in proton therapy. Ganjeh et al. analyzed the Dose Enhancement Factor (DEF) of various nanoparticles and discovered that platinum nanoparticles exhibited a higher dose enhancement coefficient, approximately 1.8 times greater than that of other nanoparticles [57]. Additionally, Zwiehoff et al. validated the enhanced radiosensitization effects of platinum nanoparticles under proton irradiation by monitoring the production of singlet oxygen [47]. Further, Zwiehoff et al. observed a significant plasmid DNA cleavage at a clinically relevant proton dose of 5 Gy by utilizing the synergetic enhancing effects between platinum nanoparticles and clinically approved stabilizing ligands [58].

#### 2.1.3. Gadolinium Nanoparticles

In addition to gold and platinum, gadolinium nanoparticles represent one of the most frequently utilized lanthanide materials, with established potential for radiosensitization in radiotherapy [59]. The simulations conducted by Wälzlein et al. indicated that protons significantly enhance local dose distribution around gadolinium nanoparticles, thereby confirming their efficacy as radiosensitizers in proton therapy, albeit with a slightly lower enhancement efficiency compared to gold and platinum nanoparticles [53]. The in vitro studies conducted by Schlathölter et al. demonstrated that the incorporation of gadolinium nanoparticles resulted in an increased incidence of DNA single-strand breaks (SSBs) and double-strand breaks (DSBs), leading to the formation of nanoscale complex lesions that substantially elevated cancer cell mortality [54]. Furthermore, Rovira et al. utilized the Geant4-DNA toolkit to simulate the nanoscale radial energy distribution of gold and gadolinium nanoparticles under proton irradiation, revealing that gadolinium nanoparticles contributed to an increase in physical dose deposition, although the enhancement was less pronounced than that observed with gold nanoparticles [60]. In 2022, Hosseini et al. conducted a comparative analysis of the dose enhancement and cytotoxic effects of three distinct nanoparticles, i.e., gold, gadolinium, and iodine, under proton beam irradiation [61]. Their findings indicate that gadolinium nanoparticles induce notable dose enhancement and increase DNA damage in cancer cells, thereby demonstrating substantial radiosensitization effects.

Additionally, gadolinium nanoparticles have been validated for use as contrast agents in magnetic resonance imaging (MRI) and as neutron capture therapy agents in recent years, attributed to their high atomic number [62,63]. Moreover, functionalized gadolinium oxide is predominantly employed as a radiosensitizer in practical applications of proton therapy rather than elemental gadolinium. For instance, gadolinium chelated within polysiloxane nanoparticles has exhibited remarkable radiosensitization effects in radiotherapy and is currently undergoing clinical trials for various tumor treatments [64,65].

#### 2.1.4. Other Metal Nanoparticles

In addition to gold, platinum, and gadolinium nanoparticles, various other metallic nanoparticles, including silver, bismuth, and iron, exhibit the potential to enhance the radiobiological effects of proton therapy [66,67,68]. The first simulation study investigating the radiation enhancement effects of various high-*Z* elements was conducted by Wälzlein et al. by utilizing the Monte Carlo tool TRAX [53]. This study demonstrated that silver nanoparticles possess a certain degree of radiosensitization in the context of proton therapy, with a local dose enhancement level comparable to that of gadolinium nanoparticles. Ganjeh et al. developed a cellular size model to evaluate the dose-enhancing effects of gold, platinum, silver, iodine, and tantalum oxide nanoparticles, focusing on the production of low-energy protons through simulation. The results indicate the following order of enhancement efficiency: platinum > gold > silver > tantalum oxide > iodine [57]. Rashid et al. examined the molecular effects of gold, platinum, bismuth, iron, and other metallic nanoparticles upon 150 MeV proton irradiation. Their findings reveal a significant radiosensitization effect associated with these nanoparticles, particularly bismuth nanoparticles, which exhibited a sensitization enhancement ratio of 4.93 and resulted in a 475% increase in singlet oxygen production in HCT116 cells compared to the control group [38].

### 2.2. Metal Oxide Nanoparticles

Certain metal oxides have demonstrated significant radiation enhancement effects in radiation therapy, surpassing the efficacy of their corresponding elemental metals. Numerous in vitro and in vivo experiments, along with simulation studies, suggest that specific metal oxides possess considerable potential for dose enhancement in proton therapy [24,69]. This section provides a detailed discussion of several metal oxides that are currently regarded as promising radiosensitizers for proton therapy.

#### 2.2.1. Hafnium Oxide Nanoparticles

Hafnium oxide is a widely utilized therapeutic agent that has been investigated in prior research for its role in promoting tissue growth around implants, serving as an effective contrast medium for computed tomography (CT) scans and functioning as a delivery carrier for targeted pharmaceuticals [70]. Its notable advantages in the radiosensitization of cancer treatment can be attributed to its excellent biocompatibility, stability, and substantial targeted accumulation at tumor sites [71]. The inaugural clinical trial assessing the radiosensitizing effects of hafnium oxide nanoparticles was conducted in 2011, involving patients with soft tissue sarcoma who received intratumoral injections of hafnium oxide nanoparticles followed by 50 Gy of external beam radiotherapy [72]. Hafnium oxide nanoparticles have been incorporated into three phase 1/2 clinical trials and one phase 2/3 trial focusing on sarcoma, head and neck squamous cell carcinoma, and liver cancer [65]. Gerken et al. performed in vitro studies to investigate the radiosensitization effects and underlying mechanisms of gold nanoparticles and metal oxide nanoparticles under photon and proton irradiation [69]. Their findings indicate that hafnium oxide nanoparticles demonstrate significant enhancements in both physical dose and chemical catalytic activity. However, there is a paucity of research regarding the biological effects of hafnium oxide in the context of proton therapy, indicating a need for further theoretical exploration and experimental validation.

#### 2.2.2. Iron Oxide Nanoparticles

Iron oxide, a widely used low-cost inorganic metal oxide, plays an important role in the realm of disease treatment due to its favorable biocompatibility and the potential for functionalization via targeted ligands, including MRI contrast enhancers and drug delivery carriers [73,74]. Recent investigations have expanded the applications of iron oxides to include roles as agents in photothermal therapy and magnetocaloric therapy and as diagnostic tools in immunotherapy [75].

The radiosensitization properties of iron oxides are particularly noteworthy as they enhance the efficacy of photon therapy by increasing radiation doses and the production of radiolysis byproducts [55,76,77]. A recent study conducted by Brero et al. examined the enhanced radiobiological effects of magnetic nanoparticles on BxPC3 pancreatic cancer cells within a synergistic treatment framework that combined proton therapy and hyperthermia [78]. The results from the in vitro experiments demonstrate that magnetic nanoparticles significantly induced radiosensitization in this combined therapy, resulting in a marked reduction in cancer cell survival, an increase in lethal DNA DSBs, and heightened production of cytotoxic ROS. This synergistic approach dramatically elevated the mortality rate of cancer cells. Additionally, Ibáñez-Moragues et al. developed zinc-doped iron oxide nanoparticles that exhibited excellent biocompatibility and stability, which enhance positron emission tomography (PET) signals and gamma-ray yield under proton irradiation [79]. This suggests that zinc-doped iron oxides not only improve dose deposition in proton therapy but also function as signal enhancers for PET and prompt gamma rays, thereby facilitating the monitoring of proton ranges within the human body during treatment.

#### 2.2.3. Titanium Oxide Nanoparticles

Titanium dioxide is classified as a low-*Z* metal oxide nanomaterial characterized by a high surface-to-volume ratio and a notable biological inertness [80]. In a comparative study of the radiosensitization effects of 22 different elemental metal oxide nanoparticles, Guerreiro et al. demonstrated that the incorporation of titanium dioxide during X-ray irradiation led to enhanced biological effects, which were attributed to an increase in the production of singlet oxygen [81]. Additionally, Gerken et al. posited that titanium dioxide generated a significant amount of reactive oxygen species when subjected to proton irradiation, and a radiosensitization effect of up to 290% at the highest experimental dose was observed. This effect surpassed that observed with hafnium oxide, tungsten oxide, and silicon oxide [69]. The nuclear reaction that occurs when titanium interacts with protons, specifically through the reaction ^48^Ti (p, x) ^48^V, results in the formation of the radioactive isotope ^48^V, which emits positrons [82]. This phenomenon may be linked to the markedly enhanced radiobiological effects of titanium dioxide under proton irradiation. Nevertheless, the precise sensitization mechanism of titanium dioxide in proton therapy remains inadequately understood and necessitates further investigation.

#### 2.2.4. Ceramic Oxide Nanoparticles

Ceramic oxide nanoparticles are ceramic compounds with nanostructures and are widely used in materials science, microelectronics, optics, and other fields [83,84]. Among these, tantalum pentoxide (Ta_2_O_5_) is recognized as a non-toxic, high-*Z* nanomaterial that, upon cellular ingestion, forms aggregates around the nucleus, resembling a protective shell [85]. This unique intracellular distribution has the potential to elicit varying dose enhancement effects when subjected to radiation exposure [86]. Brown et al. were the first to demonstrate the efficacy of Ta_2_O_5_ nanoparticles in enhancing radiation doses in vitro, revealing a significant reduction in the survival rate of 9L gliosarcoma cells following radiotherapy when these nanoparticles were internalized [87]. Following this, Engels et al. developed a Monte Carlo model based on experimental findings, which indicate that the nanoparticle shell of Ta_2_O_5_ induced highly localized physical dose enhancements during radiotherapy [88]. This enhancement was found to depend on the aggregation properties of the nanoparticles, their spatial positioning relative to the beam peak, and the energy of the photons employed. McKinnon et al. subsequently validated the nanoscale dose enhancement effects of ceramic oxide nanoparticles, specifically Ta_2_O_5_ and cerium dioxide (CeO_2_), in the context of proton therapy through Geant4 simulations, reporting average local dose enhancements of approximately 16% for Ta_2_O_5_ and 14% for CeO_2_ [89]. More recently, Ganjeh et al. quantitatively assessed the dose enhancement factor of Ta_2_O_5_ in conjunction with low-energy protons, exploring the impacts of nanoparticle size and concentration on this factor [57]. Their findings underscore the potential of Ta_2_O_5_ to augment the efficacy of proton therapy.

#### 2.2.5. Other Metal Oxide Nanoparticles

In addition to the previously mentioned metal oxide nanoparticles, various other oxides, such as tungsten oxide and silver oxide, have also demonstrated radiosensitization properties in radiation therapy. The study conducted by Gerken et al. illustrated that tungsten oxide contributes to physical dose enhancement during radiation therapy, as well as an increase in ROS resulting from surface catalysis in both photon radiation therapy and proton therapy [69]. Liu et al. provided the first evidence that silver nanoparticles can enhance the efficacy of radiation-induced cancer cell apoptosis [90]. Following treatment with silver-based nanoparticles in conjunction with radiotherapy, a significant extension in the average survival time of glioma-bearing mice was observed.

### 2.3. Other Types of Nanoparticles

Some other types of nanoparticles have also exhibited radiosensitization effects in proton therapy. For example, in vitro studies have indicated that titanium nitride (TiN) nanoparticles can enhance radiation effects by as much as 200% when subjected to proton irradiation [69]. Iodine, a substance frequently utilized as a contrast agent in diagnostic imaging due to its low toxicity, has also been shown to function as an effective radiosensitizer in proton therapy [57].

Tabbakh et al. employed Geant4-DNA to conduct a detailed microscopic analysis and perform measurements of DNA strand breaks by analyzing the effective enhancement of ^157^GdF_4_- and ^157^Gd-doped carbon nanoparticles in proton therapy [91]. In this case, the secondary alpha particles produced from nuclear reactions involving carbon and fluorine with protons (specifically, p+^12^C → 3α+p and p+^9^F → α+^16^O) result in a significant increase in both DSBs and SSBs. Moreover, the presence of ^157^Gd in nanoparticles allows for the capture of thermal neutrons, thereby mitigating the associated risks in proton therapy. This approach has the potential to substantially amplify the biological effects of proton therapy, suggesting a synergistic therapeutic strategy.

Tabbakh et al. also investigated the use of carbon nanoparticles as radiosensitizers in proton therapy [92]. The interaction between carbon ions and protons generates secondary alpha particles with high linear energy transfer (LET), while the collision of protons with carbon ions results in the recoil of target carbon ions into cancer cells. This interaction causes primary protons to decelerate and enter a high LET region due to energy loss. Simulation outcomes indicated that this combined strategy significantly enhances the relative biological effectiveness of proton therapy, with over a 300% increase in dose and thus DNA damage attributable to secondary particles. Given that carbon is a fundamental component of human physiology, carbon nanoparticles exhibit minimal biological toxicity, rendering them a safe option for tumor treatment [93].

Additionally, the potential of boron nanoparticles as radiosensitizers in proton therapy has been explored by Zavestovskaya et al. [94]. Characterization experiments assessing the effects of cell irradiation revealed that the presence of boron nanoparticles during proton irradiation resulted in elevated levels of intracellular reactive oxygen species, significantly increasing cell apoptosis and impairing cellular proliferation. This effect may be attributed to the nuclear reaction p+^11^B → α, which produces short-range α particles with high LET, thereby enhancing the biological effects of proton therapy [95]. This hypothesis was further evidenced by Wang et al. using the method of Monte Carlo simulations [96].

## 3. Mechanisms of Nanoparticle Radiosensitization

**Figure 2:**
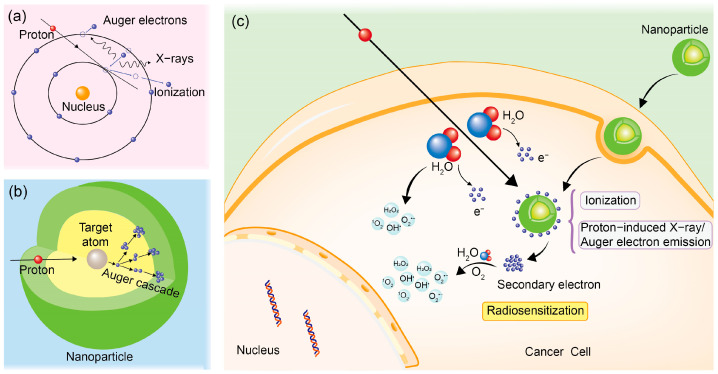
Mechanisms of nanoparticle radiosensitization in proton therapy. (**a**) Processes of ionization and emission of proton-induced X-rays and Auger electrons resulting from interactions between protons and target atoms. (**b**) Process of Auger cascade. (**c**) Illustration of increased physical dose deposition and enhanced radiolysis in cancer cell with presence of nanoparticles.

Many experimental studies and simulation processes have been carried out to explain the mechanisms of nanoparticle radiosensitization as a result of the notable and even unexpected radiosensitization effects of nanoparticles, such as gold nanoparticles, observed in proton therapy [97,98]. The mechanism can be mainly described from two aspects: the direct damage caused by the increase in physical dose and the indirect damage induced by the radiolysis processes, as illustrated in Figure 2.

### 3.1. Physical Dose Enhancement

**Figure 3:**
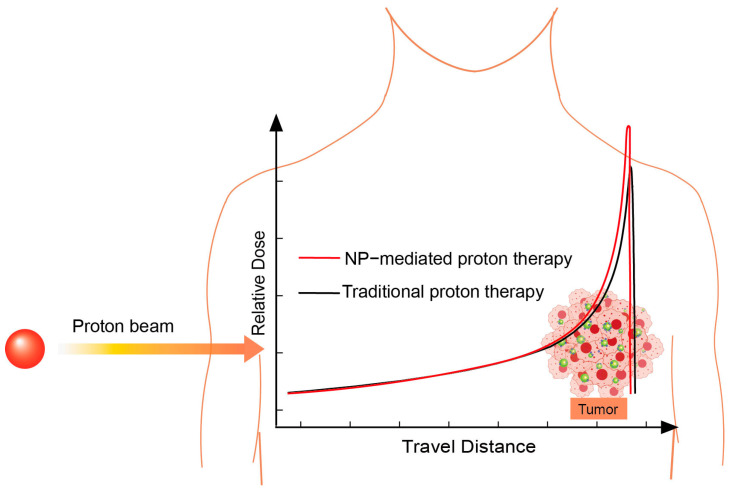
Nanoparticle-mediated enhancement of physical dose deposition of protons in comparison to conventional methods that do not utilize nanoparticles, specifically within Bragg peak region of proton therapy.

In proton therapy, protons penetrate biological tissues and interact with target atoms primarily through Coulomb interactions, especially through inelastic collisions with extranuclear electrons [12]. Due to the significant difference between protons and electrons, the energy loss of protons in each collision is relatively minor. As a result, incident protons experience a gradual loss of energy through multiple collisions with the extranuclear electrons [99]. The energy deposited per unit distance traveled by incident protons increases as their speed decreases, following an inverse relationship with the square of the proton velocity [100]. This results in a substantial energy loss per unit distance of protons towards the end of their range, leading to the formation of the Bragg peak (Figure 3). By modulating the energy of the incident protons to ensure that tumor tissue is situated within the Bragg peak region, proton therapy achieves a highly conformal dose distribution, thereby maximizing damage to cancer cells.

Nanoparticles internalized by cancer cells possess increased interaction cross-sections with protons, attributed to their high atomic number and electron density. The interaction primarily occurs through inelastic collisions between protons and the extranuclear electrons associated with the atomic constituents of the nanoparticles. When the energy gained by the extranuclear electrons exceeds their orbital binding energy, these electrons can escape the influence of the atomic nucleus, a process referred to as ionization [101]. When electrons from the inner shell of an atom are ejected, electrons from the outer shell transition inward to occupy the resulting vacancies, which leads to the emission of characteristic X-rays or Auger electrons [102] (Figure 2a). The ionized and Auger electrons may further interact with electrons from adjacent atoms, resulting in the generation of secondary electrons through multiple scattering events (Figure 2b). This process amplifies the production of low-energy secondary electrons in the nanoscale vicinity surrounding the nanoparticle [103]. These low-energy secondary electrons can subsequently interact with the surrounding biological medium, contributing to an increase in DNA damage, which is recognized as having a significantly detrimental impact on cell survival. Consequently, nanoparticles enhance the dose deposition of protons in their surrounding environment, leading to increased damage to cancer cells within the Bragg Peak region. The mechanism underlying the physical dose enhancement process of nanoparticles in proton therapy is illustrated schematically in Figure 3.

The dose-enhancing mechanism of nanoparticles in proton therapy has been extensively studied. Wälzlein et al. employed the Monte Carlo simulation tool TRAX to analyze the dose enhancement effects of nanoparticles under proton irradiation [53]. Their findings indicate that the physical dose enhancement attributed to the nanoparticles primarily resulted from the increased production of low-energy secondary electrons, predominantly generated through Auger cascades. Notably, this dose enhancement effect was confined to a limited range of a few nanometers from the nanoparticles. This observation aligns with the study conducted by Lin et al., who reported that the dose enhancement surrounding gold nanoparticles diminishes rapidly with an increasing distance from the nanoparticle surface under proton irradiation [31]. In 2022, Ahn compared the dose enhancement effects of gold nanoparticles under proton and heavy-ion irradiation, respectively. The calculatedDER within 1 nm of the gold nanoparticles indicated that both proton and heavy-ion irradiation resulted in significant dose deposition in the vicinity of the nanoparticles, with the DER decreasing rapidly as the distance from the nanoparticle surface increased [45].

Cho et al. performed a water phantom experiment to explore the dose enhancement mechanism of gold nanoparticles under proton irradiation [40]. They utilized dose detection films to measure the dose enhancement induced by high-energy secondary electrons, as well as proton-induced X-rays and gamma rays, while employing a coating in front of the detection film to obstruct secondary electrons with energies below 80 keV. The experimental results confirm that the physical dose enhancement of gold nanoparticles in proton therapy is primarily due to low-energy secondary electrons, with the average dose enhancement resulting from high-energy secondary electrons and proton-induced X-rays and gamma rays being a mere 0.1%. This finding is consistent with Dollinger’s assertion that enhancement effects based on Particle-Induced X-ray Emission (PIXE) are insufficient to account for in the observed phenomena [104]. Furthermore, Azarkin et al. assessed the impact of nuclear reactions that may occur during the interaction of protons with nanoparticles using Geant4 simulations, concluding that the effect of such nuclear reactions on nanoparticle radiosensitization is negligible [105].

### 3.2. Chemical Contributions

The indirect damage resulting from radiolysis processes is primarily facilitated by ROS, which is regarded as a significant factor contributing to the amplification of the biological effects in proton therapy. Numerous studies have substantiated this perspective through both in vitro and in vivo experiments, as well as through simulations.

Li et al. conducted a validation study on the effect of ROS on the radiosensitization of gold nanoparticles in proton therapy by utilizing the radical scavenger dimethyl sulfoxide (DMSO) in in vitro experiments [106]. The results indicate that, in the presence of DMSO, the survival fraction of cancer cells treated with nanoparticles increased from 1.3% to 25% following proton irradiation, thereby underscoring the significant role of ROS in enhancing the radiobiological effects. Following this, Gerken et al. explored the physical, chemical, and biological dose-enhancing properties of various nanoparticles through a combination of experimental and simulation approaches [69]. The introduction of hydroxyl radical scavenger DMSO markedly diminished the radiosensitization effects of the nanoparticles, thereby providing evidence that ROS-mediated indirect damage is a critical factor in the enhancement of radiation effects in proton therapy, corroborating earlier findings. Additionally, the in vitro studies conducted by Schlathölter et al. demonstrated that the presence of the radical scavenger DMSO led to a significant reduction in the incidence of DSBs and SSBs induced by platinum and gadolinium nanoparticles during proton irradiation, further confirming the predominant role of hydroxyl radicals in this context [54].

The mechanisms of ROS production are depicted in Figure 2c and can be described as follows: During interactions with protons, a large number of low-energy secondary electrons are generated in the vicinity of nanoparticles through processes such as multiple ionization and Auger cascades. These low-energy electrons subsequently engage with nearby water molecules, leading to the radiolysis of these molecules and the formation of ROS [40,107]. Within a short time (~10 ns), the ROS accumulate near the surfaces of the nanoparticles. Over time, the region with a high concentration of ROS migrates away from the nanoparticle surfaces due to the diffusion of these chemical species and an increased rate of dissociation reactions in regions with higher ROS concentrations near the nanoparticles [22]. Some ROS, such as hydrogen peroxide (H_2_O_2_) and hydroxyl radicals (•OH), are potent oxidants capable of interacting with various cellular components, including lipids, proteins, and nucleic acids. Such interactions can precipitate membrane lipid peroxidation, lysosomal instability, and mitochondrial dysfunction, ultimately triggering cellular processes such as apoptosis, necrosis, and autophagy [108,109].

Tran et al. conducted a pioneering analysis of the chemical species produced in the vicinity of gold nanoparticles using the Geant4-DNA simulation tool. Their findings revealed an enhanced radiolysis effect in the presence of gold nanoparticles alongside a time-dependent distribution of radicals [35]. In a separate study, Zwiehoff et al. evaluated five fluorescent dyes that are specific to ROS in order to assess the increased production of ROS and the influencing factors associated with high-*Z* nanoparticles under proton irradiation [47]. The results confirm a significant increase in the ROS yield generated by noble metal nanoparticles as a result of proton irradiation, a process primarily influenced by the total effective surface area of the nanoparticles.

In a combined in vitro study examining the effects of proton therapy and hyperthermia mediated by magnetic nanoparticles, 2′,7′-Dichlorofluorescein diacetate was used to detect ROS produced in BxPC3 pancreatic cancer cells upon irradiation [78]. Additionally, γ-H2AX and 53BP1 were utilized to evaluate DNA DSBs. The findings indicate that the incorporation of magnetic nanoparticles significantly enhanced ROS production and thus the incidence of DSBs compared to proton irradiation alone, resulting in an increased rate of cell death. This aligns with the cell survival outcomes observed in clonogenic assays. Cunningham et al. utilized the cytokinesis-block micronucleus (CBMN) assay to evaluate the presence of micronuclei (MNi), serving as an indicator of chromosomal breakage or complete chromosomal loss [46]. The clonogenic method was employed to assess the impact of gold nanoparticles on cellular proliferation under proton therapy. The results reveal a statistically significant reduction in cell survival rates and an increase in genetic damage in cells that were pretreated with gold nanoparticles and subsequently exposed to proton irradiation in comparison to cells subjected solely to proton irradiation. The dose enhancement of gold nanoparticles, due to the increased production of ROS, was further confirmed by Lo et al. by experimentally using the method of quantitative fluorescence measurements [110] and Zareen et al. who used theoretical simulations with TOPAS-nBio (version 1.0v), an advanced extension of the Monte Carlo simulation toolkit TOPAS (version 3.7) [111].

## 4. The Factors Influencing the Radiosensitization Effects of Protons

### 4.1. LET of Protons

The linear energy transfer (LET) of protons at specific locations within materials is directly related to the energy of the protons and exhibits an inverse relationship. Recent research has extensively examined the influence of proton energy and LET on the radiosensitization effects of nanoparticles. For instance, Tran et al. showed that the distribution of ROS produced by gold nanoparticles during proton therapy is time-dependent and inversely correlated with the energy of the incident protons [35]. Similarly, Li et al. explored the radiosensitization effects of proton beams with varying LET in conjunction with gold nanoparticles through in vitro experiments [106]. The survival curve analysis of human epidermal carcinoma A431 cells indicated that the radiobiological effects of the nanoparticles were significantly amplified when irradiated by protons with higher LET. Furthermore, Huynh et al. utilized Geant4-DNA simulations to demonstrate that as proton energy increased within the range of 0.5–25 MeV, the dose enhancement ratio surrounding the nanoparticles diminished [112]. Additionally, Hosseini et al. simulated the interactions between protons in the energy range of 0.1–20 MeV and DNA-containing targets in the presence of nanoparticles [61]. Their findings indicate that gold, gadolinium, and iodine nanoparticles enhanced the yields of SSBs and DSBs under proton irradiation. Notably, as the LET of the incident protons increased, the yield of SSBs decreased, while the yield of DSBs significantly increased. This evidence underscores that under higher-LET proton irradiation, the enhanced biological effects of nanoparticles, attributed to DNA DSBs, become increasingly significant. Mansouri et al. drew similar conclusions by performing Geant4 Monte Carlo simulations, indicating that protons with higher mass stopping power in metal nanoparticles correlate with an increase in the yields of secondary electrons [113].

### 4.2. Nanoparticle Size, Morphology, Concentration, Bio-Distribution, and Aggregation

The characteristics of nanoparticles, including their sizes, morphology, concentration, cellular bio-distribution, and degree of aggregation, are crucial in the enhanced radiation effect of nanoparticles in radiotherapy [114,115]. This section discusses the influence of these properties on the radiosensitization effects in proton therapy.

Li et al. demonstrated that both 5 nm and 10 nm gold nanoparticles can accumulate within the cytoplasm of A431 cells; however, only the 5 nm gold nanoparticles were detected on the nuclear membranes of these cells [106]. This investigation highlighted the influence of nanoparticle size on cellular uptake, identifying it as a critical factor in determining the efficacy of nanoparticle-induced radiosensitization. In the calculations performed by Huynh et al., the dose enhancement associated with gold nanoparticles was found to increase with the nanoparticle size within the range of 15 nm to 25 nm, while a gradual decrease was observed in the range of 25 nm to 50 nm [112]. Additionally, Peukert et al. found that an intermediate nanoparticle size of approximately 10–25 nm optimizes both radio-hydrolysis and dose enhancement effects since the low-energy secondary electrons produced are susceptible to self-absorption, which diminishes the enhancement effect in larger nanoparticles [19]. In contrast, Zwiehoff et al. concluded that the generation of ROS sensitized by noble metal nanoparticles is primarily governed by the total effective surface area of the nanoparticles rather than their size or mass [47]. Moreover, the study conducted by Johny et al. corroborated that the ROS produced by proton irradiation is predominantly dependent on the total number of available surface atoms on the nanoparticles, underscoring the significance of surface area effects [48].

In general, the spherical geometry of nanoparticles facilitates the release of secondary electrons generated during interactions, which results in an increased physical dose and enhanced radiosensitization effects [15]. On the other hand, the morphology of nanoparticles can affect their uptake and biocompatibility in cancer cells, thereby impacting the efficacy of radiosensitization [116]. Bartneck et al. quantitatively compared the uptake efficiency of gold nanorods and gold nanospheres in human blood phagocytes, and the results show that the uptake efficiency of nanorods was about 230 times higher than that of nanospheres with the same diameter [117]. The simulations conducted by Vácha et al. confirmed that the endocytosis of nanoparticles with sharp edges was suppressed, while spherocylindrical nanoparticles exhibited a higher propensity for internalization compared to their spherical counterparts [118]. Furthermore, Sangabathuni et al. identified the shape of gold nanoparticles as a crucial factor influencing their toxicity, biodistribution, and sequestration in Zebrafish [119]. For instance, nanorods showed more rapid uptake and clearance from the organism, whereas nanostars demonstrated slower decomposition, resulting in a prolonged retention time. A comparative study of the cellular uptake capabilities of gold nanoparticles, nanosheets, and nanorods with similar diameters and identical molecular modifications revealed that gold nanoparticles had superior cellular uptake, while nanorods exhibited the least cellular uptake. Correspondingly, their sensitization enhancement ratios were 1.62, 1.37, and 1.21, respectively, highlighting the critical influence of nanomaterial shape on radiosensitization effects [120]. Taheri et al. employed the TOPAS track structure code to evaluate the radiosensitization effects of spherical gold nanoparticles and gold nanorods by quantifying secondary electron emission and dose enhancement [121]. The results indicate that the yields of secondary electrons were similar between gold nanorods and their spherical counterparts, while the geometry of gold nanoparticles had a more pronounced effect on the emission of M-shell Auger electrons.

Rudek et al. conducted simulations to investigate the impact of various radiation types and the characteristics of gold nanoparticles (GNPs), including concentration, size, and degree of aggregation, on the radiation enhancement effects associated with these nanoparticles [122]. Their findings indicate that the dose enhancement by GNPs exhibits a linear relationship with concentration while demonstrating an inverse correlation with both the size and degree of aggregation of the nanoparticles. In a separate study, Akhdar et al. established that the dose deposition from GNPs is contingent upon the concentration of clusters at the GNP surface as the generation of secondary electrons is significantly correlated with the GNP concentration [123]. More recently, Ganjeh et al. explored the enhancement effects of nanoparticles under low-energy proton irradiation, revealing that as the concentration of nanoparticles increases, both dose deposition and the dose deposition factor are enhanced [57]. Notably, the influence of nanoparticle concentration on the sensitization effects was found to be more pronounced than that of nanoparticle size.

**Figure 4:**
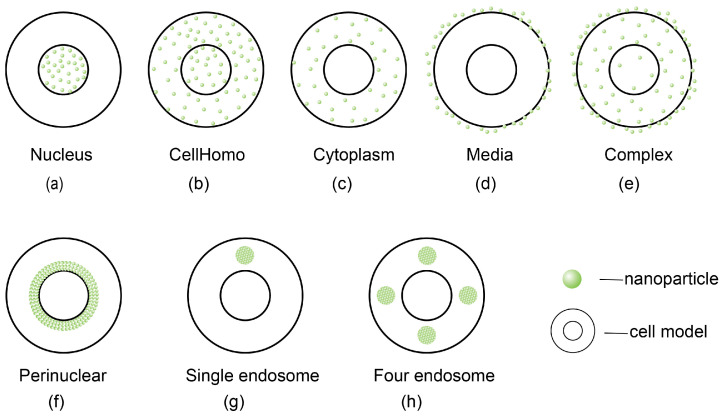
Models of nanoparticle distribution in terms of cellular geometry. (**a**) Gold nanoparticles randomly distributed in cell nucleus (nucleus model). (**b**) Gold nanoparticles randomly distributed in whole cell (CellHomo model). (**c**) Gold nanoparticles randomly distributed in cytoplasm (cytoplasm model). (**d**) Gold nanoparticles randomly distributed in extracellular media (media model). (**e**) Gold nanoparticles randomly distributed both inside cell and within extracellular media (complex model). Note that the models depicted in (**a**–**e**) were used in simulations by Lin et al. [124]. (**f**) Gold nanoparticles accumulated in perinuclear configuration (perinuclear model). (**g**) Gold nanoparticles aggregated in single compartment that sits in cytoplasm (single endosome model). (**h**) Gold nanoparticles split evenly among four spheres placed at four vertices of tetrahedron (four endosome model). Note that models depicted in (**f**–**h**) were used in simulations by Martinov et al. [125].

Lin et al. developed a biological model to investigate the radiosensitization effects of gold nanoparticles on cell survival. Their findings indicate that larger gold nanoparticles tend to allow secondary electrons to dissipate energy before reaching their surface, suggesting that smaller gold nanoparticles exhibit superior sensitization effects. Furthermore, the research highlights that the spatial distribution of gold nanoparticles within cells significantly influences radiobiological outcomes, with the most pronounced enhancement being observed when these nanoparticles were randomly distributed within the nucleus (Figure 4) [124].

Martinov et al. investigated the effects of gold nanoparticle characteristics, incident energy, and cell type on nuclear and cytoplasmic dose enhancement factors at the single-cell level using Monte Carlo simulations. Their results demonstrate a linear relationship between the concentration of gold nanoparticles and the dose enhancement factor. Importantly, the maximum dose enhancement factors for both the cytoplasm and nucleus were recorded when gold nanoparticles were accumulated in a perinuclear configuration, as depicted in Figure 3. This pattern was consistently observed across all concentrations and energies tested [125]. Subsequent research at the tumor scale further validated these findings [126]. These theoretical insights hold significant implications for the potential integration of gold nanoparticles in proton therapy, suggesting a need for additional experimental and clinical investigations in future research endeavors.

Notably, Peukert et al. conducted simulations utilizing the Geant4 Monte Carlo tool to analyze the time-dependent spatial distribution and yield of reactive species surrounding a single gold nanoparticle, two neighboring gold nanoparticles, and a cluster of gold nanoparticles, respectively, under proton irradiation [127]. The findings indicate that the presence of adjacent nanoparticles resulted in a 17% reduction in the yield of reactive species due to increased absorption, while the yield associated with nanoparticle clusters experienced a significant decrease of 60%. These results imply that the aggregation of nanoparticles within cancer cells may diminish the enhancement of biological effects, whereas a diffuse distribution of nanoparticles appears to enhance radiation sensitivity.

### 4.3. Ligand and Coating of Nanoparticles

The surface modification of gold nanoparticles has the potential to greatly enhance their biocompatibility, stability, and targeting capabilities. However, such modifications may also influence the radiosensitization effects of these nanoparticles [128]. The coatings applied to the nanoparticles can absorb low-energy photons generated during photon irradiation, functioning as a protective layer that may alter the dose enhancement effect. Furthermore, the presence of coatings and ligands can impact the chemical processes involved in the production of ROS [129,130].

Johny et al. compared the ROS amplification effects of gold nanoparticles with and without sodium citrate ligands under proton irradiation. The results indicate that the presence of ligand coatings mitigated the emission of secondary electrons generated during irradiation, thereby decreasing the production of ROS resulting from the radiolytic decomposition of water. Conversely, gold nanoparticles lacking ligands exhibited a greater specific surface area, which corresponded to a marked increase in ROS generation under proton irradiation [48].

Li et al. explored the influence of polymer coatings on the cytotoxicity and cell cycle dynamics of gold nanoparticles. Their findings reveal that the induction of cell cycle arrest by gold nanoparticles was significantly influenced by the biocompatibility of the surface coatings. Specifically, cetyltrimethylammonium bromide-modified gold nanoparticles typically induced G0/G1 phase cell cycle arrest, whereas gold nanoparticles coated with bovine serum albumin inhibited lysosomal rupture and resulted in G2/M phase arrest [131]. Peukert et al. investigated the impact of the coating thickness of individual gold nanoparticles on their radiosensitization effects during proton irradiation. Their results indicate that thicker coatings led to a reduction in dose deposition and the yield of reactive species, suggesting that the coating thickness should be minimized to achieve optimal effectiveness [19].

In contrast, Enferadi et al. examined the radiobiological effects of PEG-coated and cyclic RGDFK-conjugated ultra-small gold nanoparticles on the murine ALTS1C1 glioma cell line under various irradiation modalities, including proton, KV photon, and MV photon irradiation. They observed a significant radiosensitization effect of the functionalized gold nanoparticles across different radiation sources [36]. Additionally, Li et al. synthesized cetuximab-functionalized gold nanoparticles (Ctxb-GNPs) as targeted radiosensitizers for proton therapy. In vitro clonogenic assays demonstrated that Ctxb-GNPs accumulated in high concentrations within EGFR-overexpressing A431 cells, exhibiting enhanced biological effects under proton irradiation [132]. Similarly, the study by Zavestovskaya et al. suggested that boron nanoparticles functionalized with PEG produced a greater quantity of reactive oxygen species and displayed a more pronounced radiosensitization effect compared to their ligand-free counterparts during proton therapy [94].

## 5. Nanoparticle-Mediated Cancer Therapy: Prospective Approaches

The radiosensitization effects of nanoparticles in proton therapy still need extensive and in-depth investigations. Several novel therapeutic approaches associated with nanoparticle radiosensitization may provide valuable frameworks for future studies focused on nanoparticle-mediated proton therapy, which are discussed below.

Tumor sites typically exist within a hypoxic microenvironment due to the rapid proliferation of cancer cells, which significantly impairs the efficacy of radiotherapy, as the generation of reactive species, such as singlet oxygen, necessitates the presence of oxygen [133,134]. Chai et al. developed a radiosensitization platform that integrates photosynthetic cyanobacteria, which can release oxygen in situ, with nanoscale two-dimensional bismuth to enhance the therapeutic effect of X-ray-based therapy [135]. When subjected to combined irradiation at 660 nm and X-rays, the cyanobacteria modified the hypoxic conditions of the tumor microenvironment by producing oxygen through photosynthesis. Concurrently, the two-dimensional bismuth radiosensitizer amplified the production of ROS, resulting in a significant reduction in tumor growth in vivo. This innovative therapeutic approach, which involves the modulation of the tumor microenvironment alongside the application of radiosensitizers to augment radiobiological effects, holds potential for future applications in proton therapy with the aim of further enhancing its therapeutic efficacy.

Nanoparticles primarily accumulate in cancer cells due to the enhanced permeability and retention (EPR) effect; however, there may also be considerable accumulation in normal cells. The surface functionalization of nanoparticles facilitates their targeting of cancer cells, thereby achieving a more precise and controllable biological distribution [20,136]. For instance, the modification of nanoparticle surfaces with polyethylene glycol (PEG) can enable them to evade detection by the immune system and regulate their surface charge, which, in turn, affects their biological distribution [137]. Furthermore, the specific interaction between ligands on the nanoparticle surface and receptors that are overexpressed on cancer cell surfaces allows for the active targeting of these cells [138,139]. For example, folate-modified gold nanoparticles can specifically target the elevated folate receptors present in cancer cells, while antibody-coated gold nanoparticles can selectively bind to overexpressed receptors on the surfaces of these cells. Such nanoparticles, possessing active targeting capabilities, hold significant promise as effective radiosensitizers in proton therapy.

In addition, the synergistic application of radiation therapies with adjunctive therapies presents significant potential for therapeutic advancement. For instance, Fathy et al. synthesized chitosan-capped gold nanoparticles (CS-GNPs-dox) that were loaded with the chemotherapeutic agent Doxorubicin, subsequently conducting chemo-radiotherapy enhanced by gold nanoparticle sensitization [140]. The findings from biological experiments demonstrate that these functionalized gold nanoparticles markedly improved the therapeutic efficacy against tumors by promoting increased DNA DSBs and inducing cell necrosis. This integrative approach of radiotherapy and chemotherapy facilitates the targeted delivery of chemotherapeutic agents to cancer cells while simultaneously augmenting the radiosensitization effects of radiation therapy. In another remarkable example, Gholami et al. investigated the enhanced radiation damage effects achieved by labeling radioactive isotopes onto superparamagnetic iron oxide nanoparticles. The dose enhancement observed from radiotherapy utilizing isotope-labeled nanoparticles was significantly greater than that achieved with conventional radiotherapy methods [141]. Collectively, these findings underscore the potential of synergistic therapy in enhancing the RBE of proton therapy.

## 6. Conclusions

In summary, this review explores recent advances in the investigation of the radiosensitizing effects of nanoparticles in the context of proton beam irradiation, which presents significant potential for application in proton-based cancer treatment. A systematic summary and classification of nanoparticles that demonstrate notable radiosensitization properties are provided alongside an elucidation of the mechanisms by which these nanoparticles enhance the efficacy of radiotherapy. Additionally, this review addresses various factors that may influence the radiosensitization capabilities of nanoparticles within the framework of proton therapy. The exploration of the radiosensitizing effects of nanoparticles in proton therapy has attracted significant attention in recent years, resulting in a substantial volume of ongoing research. However, the practical implementation and clinical translation of nanoparticles in therapeutic contexts require further simulation and experimental data to substantiate their efficacy, underscoring the necessity for continued investigations.

## Data Availability

Not applicable.
